# Mining from transcriptomes: 315 single-copy orthologous genes concatenated for the phylogenetic analyses of Orchidaceae

**DOI:** 10.1002/ece3.1642

**Published:** 2015-08-20

**Authors:** Hua Deng, Guo-Qiang Zhang, Min Lin, Yan Wang, Zhong-Jian Liu

**Affiliations:** 1 State Key Laboratory of Tree Genetics and Breeding, Key Laboratory of Tree Breeding and Cultivation of State Forestry Administration, Research Institute of Forestry, Chinese Academy of ForestryBeijing, 100091, China; 2 Shenzhen Key Laboratory for Orchid Conservation and Utilization, The National Orchid Conservation Center of China and The Orchid Conservation and Research Center of ShenzhenShenzhen 518114, China; 3 The Center for Biotechnology and BioMedicine, Graduate School at Shenzhen, Tsinghua UniversityShenzhen 518055, China; 4 College of Forestry, South China Agricultural UniversityGuangzhou 510642, China

**Keywords:** Orchidaceae, phylogeny, RNA sequences, single-copy orthologous genes

## Abstract

Phylogenetic relationships are hotspots for orchid studies with controversial standpoints. Traditionally, the phylogenies of orchids are based on morphology and subjective factors. Although more reliable than classic phylogenic analyses, the current methods are based on a few gene markers and PCR amplification, which are labor intensive and cannot identify the placement of some species with degenerated plastid genomes. Therefore, a more efficient, labor-saving and reliable method is needed for phylogenic analysis. Here, we present a method of orchid phylogeny construction using transcriptomes. Ten representative species covering five subfamilies of Orchidaceae were selected, and 315 single-copy orthologous genes extracted from the transcriptomes of these organisms were applied to reconstruct a more robust phylogeny of orchids. This approach provided a rapid and reliable method of phylogeny construction for Orchidaceae, one of the most diversified family of angiosperms. We also showed the rigorous systematic position of holomycotrophic species, which has previously been difficult to determine because of the degenerated plastid genome. We concluded that the method presented in this study is more efficient and reliable than methods based on a few gene markers for phylogenic analyses, especially for the holomycotrophic species or those whose DNA sequences have been difficult to amplify. Meanwhile, a total of 315 single-copy orthologous genes of orchids are offered and more informative loci could be used in the future orchid phylogenetic studies.

## Introduction

Orchidaceae is the largest and most diverse family of angiosperms, with approx. 25,000 species in 870 genera (Cribb et al. 2003; Swarts and Dixon 2009). The orchids are distributed among tropic, subtropic, and temperate regions but have primarily been observed in the tropics, particularly the Neotropics (Hagsater et al. 1996; Swarts and Dixon 2009). Apart from the well-known member Vanilla, a number of genera, such as *Dendrobium*, *Paphiopedilum*, *Cypripedium*, *Vanda,* and *Cymbidium*, are of great ornamental value based on the amazing forms and colorful inflorescences of these plants, which have gradually become the most important economical crops in many countries of South-East Asia. In addition to economic importance, orchids are also of great evolutionary and ecological significance. The evolution of orchids dates back to Charles Darwin, and numerous researchers have followed in his footsteps, using orchids as model organisms to examine the evolution and suitability of some plants to various environments worldwide. Moreover, most wild orchid species experience overexploitation and exist in threatened communities, reflecting the destruction of habitats and prosperity of industries. Orchid phylogeny is the primary foundation in various fields, such as evolution, ecology, molecular biology, and physiology.

The phylogenic analyses of the relationships of species inferred from primitive or advanced groups, positioning the species in the correct phylogenetic frame, could insightfully reveal evolutionary patterns and mechanisms (Soltis and Soltis 2003). Traditionally, orchid phylogenies have been morphologically based, particularly on the characteristics of the flowers (Dressler and Dodson 1960; Ackerman and Williams 1980; Dressler 1981, 1993; Burns Balogh and Funk 1986). This classical phylogeny requires professional familiarity with all of the morphological characteristics of the groups and their relatives to identify and classify them accordingly. Experts with rich experience in certain genera or species might be required, as is the case for Orchidaceae, which show a wide morphological range. Different classification criteria considered by different people might lead to inconsistent results, generating controversial standpoints. Morphology-based phylogenies are susceptible to the subjective factors considered by the researcher. In addition, some genera cannot be positioned correctly when only assessed based on morphology and anatomy. For instance, the primitive Apostasioideae subfamily, previously identified as a separate family, was not initially considered as an orchid, as this plant showed no close resemblance to other orchids (Kristiansen et al. 2001).

The widely sampled molecular study of Orchidaceae was first performed in 1994 (Chase et al. 1994). The molecular methods applied in phylogenic analyses have greatly advanced our comprehension of orchids relationships (Freudenstein et al. 2004). Current phylogenies tend to use multiple markers, combining plastid genes with a few low-copy nuclear genes, to improve bootstrap values and reconstructed a relatively reliable orchid phylogeny (Pridgeon et al. 2001a,b, 2014; Cameron 2009; Chase et al. 2015). In the last 20 years, considerable effort has been devoted to the orchid phylogeny (Pridgeon et al. 1999, 2001a,b, 2003, 2005, 2009, 2014) and the general phylogeny of Orchidaceae is constructed as it is today, hence contributing greatly to our understanding of orchid evolution.

The key of these molecular procedures is selection of appropriate marker genes (Igea et al. 2010; Patwardhan et al. 2014) and the generation of amplification products. However, the selection of suitable marker genes is difficult, as some markers are particular to a certain taxonomic level (Capella-Gutierrez et al. 2014) with insufficient resolution for the examination of the phylogeny of other levels. Although suitable markers have been selected, the amplification of corresponding DNA fragments is typically PCR based and therefore labor intensive or even difficult to amplify for some species. Furthermore, Orchidaceae, being as the most diverse plant family on earth (Dressler 1981), has many holomycotrophic species with degenerated plastid genome (Rothacker 2007). The placement of these holomycotrophic taxa remains problematic, due to its great difficulty in amplifying plastid DNA loci (Cameron et al. 1999; Cameron 2004). Therefore, a more rapid and efficient method, which could increase the throughput of library analyses and, most importantly, confirm the placement of holomycotrophic taxa, is needed for Orchidaceae.

Illumina second-generation sequencing (RNA-Seq) is rapidly emerging as an efficient platform for quantitative transcriptome profiling. Offering enormous single-copy nuclear genes at one time (Wang et al. 2008), RNA-Seq data have facilitated the highly efficient and reliable construction of orchid phylogeny. Using concatenated single-copy orthologous genes to resolve phylogenic relationships could provide a more reliable evolutionary framework than using several specific genes, as biparental inheritance and large numbers of informative sites conferred by low-copy protein-coding nuclear genes show many advantages (Zhang et al. 2012). The conserved single-copy nuclear genes identified through genome comparison have been used to improve phylogenetic resolution (Duarte et al. 2010; Zhang et al. 2012). Here, we demonstrated that RNA-Seq data are highly useful for the phylogeny construction of Orchidaceae, including species that are difficult to position. We proposed that with reduced cost and increased application, transcriptome-based phylogeny construction is an attractive option for Orchidaceae.

## Materials and Methods

### Ethics statement

No specific permits were required for the described field studies.

### Selection of representative species

Apostasioideae is a primitive subfamily of orchids with only two genera, *Neuwiedia* and *Apostasia*, which include herb species with woody bases and three stamens (Kocyan et al. 2004; Wu et al. 2009). A total of 17 species have been accepted in this subfamily, which is primarily located in South-East Asia (“WCSP (2015). World Checklist of Selected Plant Families. Facilitated by the Royal Botanic Gardens, Kew. Published on the Internet; http://apps.kew.org/wcsp/”). *Neuwiedia malipoensis* is a new species from Yunnan, China.

The subfamily Vanilloideae is comprised of 24 genera and approx. 185 species, which are monandrous with a pantropical distribution. *Vanilla shenzhenica* is an endemic species of China and is normally observed climbing along trees or cliffs in forests or valleys below an altitude of 300 m (Wu et al. 2009).

Cypripedioideae is comprised of five genera and approx. 180 species, which have two fertile stamens and are widely distributed through the Northern Hemisphere (Wu et al. 2009). These species are commonly known as slipper Orchids, reflecting the special pouch-like labellum structure of these plants. *Paphiopedilum* and *Cypripedium* are the richest genera in this subfamily; therefore, we selected two representative species from these two genera. *Paphiopedilum armeniacum* is terrestrial or lithophytic, and the leaves of these plants are thick and leathery. *Cypripedium singchii* is rare and endemic in China, preferring mid-shaded to deep shaded habitats and occurring in thickets and shrubby slopes.

Orchidoideae has approximately 192 genera and 3630 species (Wu et al. 2009). The species of this subfamily are monandrous and widely distributed in north temperate zones and tropical areas. *Hemipilia forrestii*, a representative species, has erect and one-leaved stems and lives on rocky slopes.

Epidendroideae is the largest and most diverse subfamily in Orchidaceae with approximately 600 genera and 18,000 species distributed worldwide, except for Antarctica (Wu et al. 2009; Pridgeon et al. 2014). The showy flowers of some of these plant species are important parents in hybridization. Despite the cosmopolitan distribution of these plants, most species are tropical epiphytes, typically with succulent and swollen leaves or stems, likely associated with crassulacean acid metabolism plants (Winter et al. 1983; Silvera et al. 2005; Griffiths et al. 2008; Barrera Zambrano et al. 2014). Given the number of diversified species in Epidendroideae, such as terrestrial and epiphytic, sympodial and monopodial, and achlorophyllous and autotrophic orchids, we selected five species to represent this large subfamily. *Gastrodia elata* is an achlorophyllous and a holomycotrophic orchid plant, feeding in symbiosis with fungi. It is difficult to amplify the plastid genes from *Gastrodia* and other achlorophyllous taxa (Cameron et al. 1999; Cameron 2004), as the plastids of this genome are degenerated (Rothacker 2007). Previous phylogenic analyses have roughly placed *Gastrodia* (Chase et al. 2003; Górniak et al. 2010) at the base of Epidendroideae with low bootstrap values; thus, the position of this subfamily remains problematic. *Cymbidium sinense* is well known for strongly fragrant flowers. *Phalaenopsis equestris*, *Gastrochilus calceolaris,* and *Holcoglossum amesianum* are advanced orchids of the subfamily Aeridinae.

### RNA extraction and transcriptome sequencing

Total RNA was extracted from leaf and flower tissues samples obtained at The National Orchid Conservation Center of China (NOCC) using the Spectrum™ Plant Total RNA Kit (Sigma; Saint Louis, MO, USA). The transcriptome library construction and sequencing were performed according to the procedures of Peng's work (Peng et al. 2012). After isolation with beads and Oligo (dT), fragmentation buffer was added and the mRNA was degraded into short segments. The short segments were used as templates, and the first-strand cDNA was produced using random hexamer primers. Subsequently, the second-strand cDNA was synthesized after adding buffer, dNTPs, RNase H, and DNA polymerase I. The short fragments were purified using the QiaQuick PCR extraction kit, terminally repaired with the addition of EB buffer and poly (A), and successively connected using sequencing adapters. Subsequently, agarose gel electrophoresis was used to select fragments of ideal lengths. Suitable fragments were amplified after 15 cycles of PCR, and the related libraries were constructed. Moreover, the libraries were sequenced using Illumina HiSeq™ 2000 according to the manufacturer's instructions (Illumina Inc., San Diego, CA, USA). HiSeq Control Software (HCS v1.1.37) with RTA (v1.7.45) was used for the management and execution of HiSeq™ 2000 experiment runs.

### *De novo* assembly and Unigene prediction

Transcriptome *de novo* assembly was performed using the short reads assembly program Trinity (Grabherr et al. 2011) (parameter: min_glue = 2, V = 10, edge-thr = 0.05, min_kmer_cov = 2, path_reinforcement_distance = 75, group_pairs_distance = 250). It first links reads with a certain length of overlap to shape longer fragments without any gap that are called contigs. The reads are subsequently used to align back to contigs. Using paired-end reads, contigs from the same transcript and the distances between are detected. Next, the contigs to scaffolds are linked using N to represent unknown sequences between two contigs. Paired-end reads are used for gap filling of scaffolds. Such sequences are defined as Unigenes. When multiple samples from the same species are sequenced, the Unigenes from the assembly of each sample are further processed through sequence splicing and redundancy removing using sequence clustering software to acquire nonredundant Unigenes of long lengths. In the final step, blastx alignments (*e*-value < 0.00001) between Unigenes and protein databases, such as the NCBI nonredundant database (NR), Swiss-Prot (http://www.uniprot.org), KEGG (http://www.genome.jp/kegg/), and COG (http://www.geneontology.org), were performed, and the best aligning results were used to determine the sequence direction of the Unigenes.

### Gene family identification

**Figure 1:**
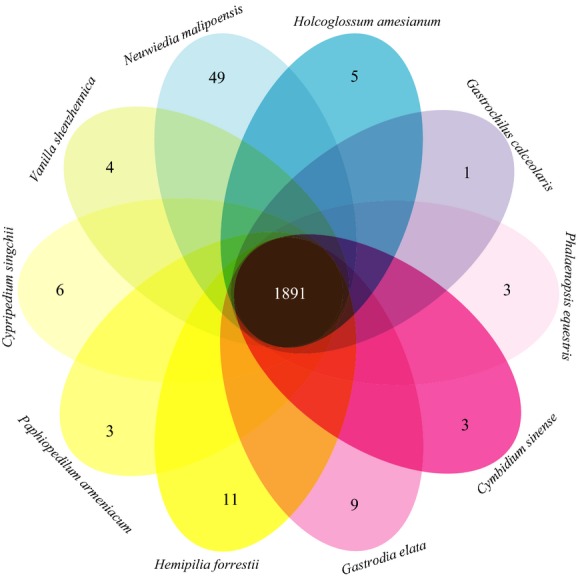
Venn diagram of the shared orthologous gene families among 10 orchids. In total, 1891 gene families (containing 315 single-copy gene families) were shared among 10 orchids. The numbers of the specific gene cluster of each species are shown in ellipses.

The following pipeline was used to cluster individual genes into gene families: (i) all-to-all blastp was used to align all protein sequences with an *e*-value of 1*e*-5, and (ii) the gene families (Fig.1) were clustered using OrthoMCL software (Li et al. 2003).

### Phylogenetic reconstruction

MUSCLE (Edgar 2004) was used to independently obtain multisequence alignments from 315 single-copy gene families. All alignments were concatenated to a “supergene” for phylogenetic analyses using the optimality criteria of maximum likelihood and Bayesian inference implemented in PhyML (Guindon et al. 2010) and BayesPhylogenies (Pagel and Meade 2004), respectively. The maximum likelihood tree was calculated using a GTR + CAT model and *Neuwiedia malipoensis* as the out-group species. For the Bayesian tree, we used the GTR model to construct a supergene with one million generations to produce a robust tree.

## Results

To discover accurate and well-supported phylogenies, transcriptome (RNA-Seq) data were obtained from 10 orchid species covering five subfamilies with various morphological characters (containing flower shapes, structure, color, etc.) and distribution in diverse habitats, including terrestrial, epiphytic, lithophytic, or holomycotrophic orchids (Wu et al. 2009). After sequencing using Illumina HiSeq 2000, we constructed non-normalized cDNA libraries for each species and subsequently obtained 20 million 90-bp paired-end cDNA sequence reads. The sequenced reads were *de novo* assembled using Trinity software (Grabherr et al. 2011), and only contigs of lengths >150 bp were retained for further analyses. Moreover, individual genes were clustered into gene families. Overall, 10 orchids shared 1891 gene families (Fig.1), 315 of which are single-copy gene families (Table S1). These orthologous genes were concatenated into a supergene and applied for phylogenetic construction.

**Figure 2:**
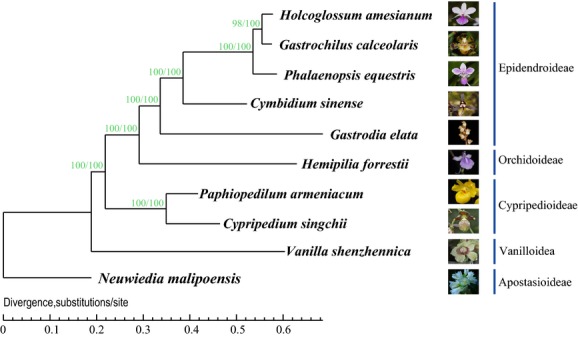
A phylogram of the best ML and BI tree constructed based on 315 single-copy orthologous genes. The phylogenetic relationships of the five Orchidaceae subfamilies with the bootstrap values. Each species with flower pictures on the right to represent their subfamilies.

Based on 315 single-copy genes with concatenated alignments, the maximum likelihood (ML) tree generated using PhyML software (Guindon et al. 2010) exhibited the robust relationships of the orchid family with almost 100% bootstrap values on all nodes. Moreover, the Bayesian inference (BI) phylogeny predicted using BayesPhylogenies (Pagel and Meade 2004) was completely consistent with the ML tree. Both phylogenetic trees with ML and BI methods supported the same topology (Fig.2), suggesting that all taxa of Orchidaceae were represented by sufficient sequence information for reliable placement.

We set *Neuwiedia malipoensis* (Apostasioid) as the out-group species. The transcriptome phylogeny (Fig.2) was built using model-based analytical methods, supporting *Vanilla shenzhenica* as the basal clade after diverging from *Neuwiedia,* followed by the clade formed by Cypripedioideae, Orchidoideae, and Epidendroideae. Epidendroideae is located at the top of the phylogenetic tree with the bulk of the taxa in the family: *Gastrodia elata*, *Cymbidium sinense*, *Phalaenopsis equestris*, *Gastrochilus calceolaris,* and *Holcoglossum amesianum*, and the latter three species shape the subclade Aeridinae as higher Epidendroids (Cameron et al. 1999; Kocyan et al. 2004). *Gastrochilus calceolaris* is positioned at the basal nodes of Epidendroideae. The phylogeny is largely consistent with previous results (Chase et al. 2003; Górniak et al. 2010) with 100% bootstrap values at almost all nodes.

## Discussion

Brieger suggested five Orchidaceae subfamilies (Brieger 1958), namely Apostasioideae, Vanilloideae, Cypripedioideae, Orchidoideae, and Epidendroideae, based on morphological and embryological analyses. Recent molecular analyses offer a more reliable phylogeny than traditional classification, and extensive studies have focused on the selection of reliable plastid or nuclear genes to construct orchid phylogenies (Cameron et al. 1999; Chase et al. 2003; Cameron 2004; Freudenstein et al. 2004; Neubig et al. 2008; Górniak et al. 2010), which typically support Apostasioideae as the basic clade in all orchid branches (Chase et al. 2003; Górniak et al. 2010). Therefore, *Neuwiedia malipoensis* (Apostasioid) was set as the out-group species in the tree presented herein.

Described indefinitely as near the basal nodes of the orchid tree, the positions of Cypripedioideae and Vanilloideae have been variable (Cameron 2004; Górniak et al. 2010), whereas Orchidoideae and Epidendroideae are consistently derived from a sister clade located at the top of the tree. Although some nodes remain problematic with low bootstrap values, the relationship of the five subfamilies is generally accepted as (Apostasioideae (Vanilloideae (Cypripedioideae (Orchidoideae (Epidendroideae))))) (Chase et al. 2003; Freudenstein et al. 2004; Górniak et al. 2010). Ten species were represented based on the constructed “supergene”, concatenated using 315 single-copy genes, and analyzed for orchid transcriptome phylogeny. Both of the phylogenetic inference methods ML and BI support the same topology (Fig.2) and are completely consistent with the foregoing relationship. The transcriptome phylogeny presented herein not only confirms previous results (Chase et al. 2003; Górniak et al. 2010) but also provides more robust confidence with almost 100% bootstrap values (except for the 98% on the top of the tree).

Notably, *Gastrodia* is a holomycotrophic species without chlorophyll. Holomycotrophic plants generally evolved faster than their nonholomycotrophic relatives in molecular sequences for all three genomes, that is, the mitochondrial, nuclear, and chloroplast genomes (Rothacker 2007; Bromham et al. 2013). Due to the degenerated plastid genome (Rothacker 2007), several attempts to amplify plastid genes from the holomycotrophic orchid *Gastrodia* and other achlorophyllous taxa have failed (Cameron et al. 1999; Cameron 2004). Although most orchid phylogeneticists have confirmed that *Gastrodia* was located at the base of Epidendroideae (Chase et al. 2003; Górniak et al. 2010), the bootstrap values of previous phylogeny were quite low, and the position of this subfamily remains problematic. The Orchidaceae transcriptome phylogeny presented herein provides 100% support for the position of *Gastrodia*. Despite this high bootstrap value, the specific position of *Gastrodia* is still not confirmed in this study, due to the lack of members of tribe Neottieae. It seems probable that *Gastrodia* is a close relative of some member of higher Epidendroideae unsampled here. More orchid genera are needed to sample in the further work for confirmation of the specific placement of *Gastrodia*. Even so, this study offers a model approach for the phylogenic analysis of holomycotrophic species or those whose DNA sequences have been difficult to amplify, which has been problematic for a long time in orchid phylogenetics.

## Conclusion

A more robust phylogeny of Orchidaceae was reconstructed using 315 single-copy orthologous genes extracted from RNA-Seq data. ML and BI phylogenies support the same topology with almost 100% bootstrap values on all nodes, and these results are consistent with previous phylogenetic trees, confirming that this transcriptome phylogeny represents the robust relationships of the orchid family. As a holomycotrophic plant, the position of *Gastrodia* is controversial. The transcriptome phylogenic analysis placed *Gastrodia* with high bootstrap value, suggesting this organism as an adequate representative of holomycotrophic plants and so forth. Therefore, using RNA-Seq data to reconstruct Orchidaceae phylogeny is a more rapid, reliable, and efficient technique than current methods based on a few gene markers, especially for the holomycotrophic species or those whose DNA sequences have been difficult to amplify. Meanwhile, a total of 315 single-copy orthologous genes of orchids are offered and more informative loci could be used in the future orchid phylogenetic studies.

## Data Accessibility

Data has been deposited in the Dryad Digital Repository: http://dx.doi.org/10.5061/dryad.qm30m

## Conflict of Interests

The authors have declared that no competing interests exist.

## Supporting Information

Table S1 . Gene IDs of 315 single-copy families of 10 orchids.
